# Integration of System Biology Tools to Investigate Huperzine A as an Anti-Alzheimer Agent

**DOI:** 10.3389/fphar.2021.785964

**Published:** 2021-12-13

**Authors:** Pukar Khanal, Farshid Zargari, Bahareh Farasati Far, Dharmendra Kumar, Mogana R, Yasir K. Mahdi, Najwan K. Jubair, Shailendra K. Saraf, Parveen Bansal, Ranjit Singh, Malarvili Selvaraja, Yadu Nandan Dey

**Affiliations:** ^1^ Department of Pharmacology and Toxicology, KLE College of Pharmacy Belagavi, KLE Academy of Higher Education and Research (KAHER), Belagavi, India; ^2^ Pharmacology Research Center, Zahedan University of Medical Sciences, Zahedan, Iran; ^3^ Department of Chemistry, Faculty of Science, University of Sistan and Baluchestan, Zahedan, Iran; ^4^ Department of Chemistry, Iran University of Science and Technology, Tehran, Iran; ^5^ Department of Pharmaceutical Chemistry, Laureate Institute of Pharmacy, Kangra, India; ^6^ Faculty of Pharmaceutical Sciences, UCSI University, Kuala Lumpur, Malaysia; ^7^ Faculty of Pharmacy, BBDNIIT, Lucknow, India; ^8^ University Centre of Excellence in Research, Baba Farid University of Health Sciences, Faridkot, India; ^9^ AVIPS, Shobhit University, Saharanpur, India; ^10^ Department of Pharmacology, Dr. B.C. Roy College of Pharmacy and Allied Health Sciences, Durgapur, India

**Keywords:** Alzheimer’s disease, huperzine A, system biology, donepezil, ligand–receptor interactions

## Abstract

**Aim:** The present study aimed to investigate huperzine A as an anti-Alzheimer agent based on the principle that a single compound can regulate multiple proteins and associated pathways, using system biology tools.

**Methodology:** The simplified molecular-input line-entry system of huperzine A was retrieved from the PubChem database, and its targets were predicted using SwissTargetPrediction. These targets were matched with the proteins deposited in DisGeNET for Alzheimer disease and enriched in STRING to identify the probably regulated pathways, cellular components, biological processes, and molecular function. Furthermore, huperzine A was docked against acetylcholinesterase using AutoDock Vina, and simulations were performed with the Gromacs package to take into account the dynamics of the system and its effect on the stability and function of the ligands.

**Results:** A total of 100 targets were predicted to be targeted by huperzine A, of which 42 were regulated at a minimum probability of 0.05. Similarly, 101 Kyoto Encyclopedia of Genes and Genomes pathways were triggered, in which neuroactive ligand–receptor interactions scored the least false discovery rate. Also, huperzine A was predicted to modulate 54 cellular components, 120 molecular functions, and 873 biological processes. Furthermore, huperzine A possessed a binding affinity of −8.7 kcal/mol with AChE and interacted within the active site of AChE *via* H-bonds and hydrophobic interactions.

## Introduction

Alzheimer’s disease (AD) is one of the neurodegenerative pathogeneses that majorly affect geriatric subjects, characterized by increased confusion and impaired cognitive function, and difficulty in learning and organizing thoughts (Bondi et al., 2017). Compromised cholinergic function, uplifted expression of β-amyloid, and unregulated oxidative stress are considered as the major hypotheses (Bondi et al., 2017; Grewal et al., 2021) *via* which the AD pathogenesis propagates. Also, deregulation of multiple neurotransmitter synapses and pathways has been traced for AD progression (Francis, 2005). Also, AD pathogenesis has been categorized as polygenic (Bird, 2008) due to the involvement of multiple genes in its progression.

One of the well-accepted approaches to manage AD includes acetylcholinesterase (AChE) inhibition, *via* which donepezil has been developed. However, donepezil is associated with multiple side effects like nausea, vomiting, weight loss, frequent urination, and muscle cramps. Also, other anti-Alzheimer’s drugs like *N*-methyl-d-aspartate antagonists, nicotine receptor agonists, peroxisome proliferator-activated receptor-γ agonists, and 5-hydroxytryptamine modulators are linked with multiple side effects (Casey et al., 2010). This underlines the necessity of identifying new therapeutic agents for the pharmacotherapy of AD.

Huperzine A is a naturally occurring sesquiterpene alkaloid, obtained from the extract of firmoss *Huperzia serrata*. In China, *H. serrata* is used in treating fever, swelling, and blood disorders. Additionally, it also exhibits neuroprotective properties and is under investigation as a possible agent to deal with the AD (PubChem, 2004). Furthermore, huperzine A has been reported for its neuroprotective effect (Wang and Tang 2005) and the possibility of AChE inhibition (Pohanka 2014). However, it is yet to be examined for its potentiality in regulating the multiple proteins and pathways based on the principle “a single compound can regulate multiple proteins and trigger various pathways related to them” (Khanal et al., 2021). Thus, it becomes imperative to investigate if huperzine A can trigger multiple proteins, so as to reveal its complex pharmacological spectra linked to AD. Hence, the present study aimed to investigate huperzine A as a possible candidate against AD, *via* the concept of “multi-protein pathways” interaction, by utilizing a series of system biology tools.

## Materials and Methods

### Gene Ontology Analysis

Canonical SMILES of huperzine A and donepezil were retrieved from the PubChem (https://pubchem.ncbi.nlm.nih.gov/) database and queried in SwissTargetPrediction (Daina et al., 2019; http://www.swisstargetprediction.ch/) to predict the possible targets. The regulated targets were matched with the recorded AD targets (DisGeNET entry: C0002395; https://www.disgenet.org/home/) and visualized using venny 2.1 (Oliveros, 2007-2015; https://bioinfogp.cnb.csic.es/tools/venny/). Furthermore, these targets were enriched in STRING (Szklarczyk et al., 2019; https://string-db.org/) to identify the possibly modulated cellular components, molecular functions, and biological processes. Also, huperzine A-regulated pathways were identified using the Kyoto Encyclopedia of Genes and Genomes (KEGG; https://www.genome.jp/kegg/) database.

### Molecular Docking

In target prediction, huperzine A was predicted to possess the highest probability to regulate AChE, at a probability >0.9. Hence, it was considered further for molecular docking.

#### Ligand Preparation

The 3D structures of huperzine A and donepezil were retrieved from PubChem database in .sdf format and converted into .pdb format using Discovery Studio 2020 (Dassault Systèmes BIOVIA (2020) Discovery Studio, 2020; Dassault Systèmes, San Diego; https://discover.3ds.com/discovery-studio-visualizer-download). The energy of both the ligands was minimized using mmff94 force field (Halgren 1996) under conjugate gradient algorithm, using Open Babel at PyRx (https://pyrx.sourceforge.io/) ver*.* 0.8. Then, the molecules were converted into .pdbqt format, which was later used for docking as the ligand.

#### Macromolecule Preparation

The 3D crystallographic structure of AChE (PDB: 4EY7; Cheung et al., 2012) was retrieved from the RCSB protein data bank (https://www.rcsb.org/), which was in a complex with water molecules and other hetero-atoms. These pre-complex hetero-atoms or molecules were removed using Discovery Studio 2020 and saved in .pdb format, which was later used as the target for docking.

#### Ligand–Protein Docking

Both the ligands were docked against AChE using AutoDock Vina (Trott and Olson, 2010) by setting the grid box as center x, y, z = −2.895, −40.11, 30.76 and size x = 59.75, 61.24, 72.51. After docking, 10 different confirmations of both the ligands were obtained. The ligand with the minimum binding energy was chosen to visualize the ligand–protein interactions using Discovery Studio 2020.

### Molecular Dynamics Simulations

To explore the part of the ligand responsible for layout within the active site of AChE and potential inhibitory activity, the best docking pose of both the ligands was chosen. Each of the selected ligand pose was initially simulated for 150-ns molecular dynamics (MD) simulations, in explicit water. The parameters for the ligands were then extracted from the general amber force field (Wang et al., 2004) using the Amber Tools package (https://ambermd.org/AmberTools.php). The partial charge on each atom was treated with a restrained electrostatic potential charge (Vanquelef et al., 2011). The conversion of AMBER topology and coordinate file was done with AnteChamberPYthon Parser interfacE (Sousa da Silva and Vranken, 2012; ACPYPE; https://www.bio2byte.be/acpype/). All the systems were fully solvated in the TIP3P water model, and ions were added to neutralize the net charge on the system. In the present study, the Gromacs 2020.2 package was utilized for the MD simulations (Van Der Spoel et al., 2005; http://www.gromacs.org/), and the Amber99sb-ildn force field was used to treat AChE (Beauchamp et al., 2012). Furthermore, periodic boundary conditions were applied in XYZ coordinate. Additionally, the energy on each system was minimized using steeped descent algorithm. The temperature equilibration of the systems was conducted in NVT ensemble, with 0.1-ps coupling constant, in a modified Berendsen thermostat (Berendsen et al., 1984) for 500 ps to maintain the temperature at 300 K. Also, 1-ns pressure equilibration was applied in NPT with the Berendsen Barostat (Berendsen et al., 1984), with a 2.0-ps coupling constant at 1 bar. The production was run in NPT, and a time step of 2 fs was followed, till the restraint was removed. The Particle Mesh Ewald (Darden et al., 1993) method interaction was used for the long-range electrostatic study. Furthermore, a cutoff of 12 Å for long-range and LINKS algorithms for H-bond constraints were applied (Hess et al., 1997). The equilibration and production run H-bond outlines between the ligand and AChE were calculated using the g H-bond module of Gromacs, as detailed previously (Zargari et al., 2018).

#### Principal Component and Dynamic Cross-Correlation Map Analysis

Principal component analysis (PCA) reveals the dominant modes in the motion of molecules by exploiting MD trajectories (Amadei et al., 1993; Amadei et al., 1996). This includes the elimination of the rotational and translational motion of the molecule using the least square fit to the reference structure. Furthermore, a covariance matrix is produced by a linear transformation of Cartesian coordinate space and also diagonalized to generate a set of eigenvectors to indicate the direction of the motion of the molecule. The eigenvalue corresponding to each eigenvector represents the energy contribution of that particular component to the motion. Projection of the trajectory on a particular eigenvector highlights the time-dependent motions that the components perform in a particular vibrational mode. The time average of the projection shows the contribution of components of the atomic vibrations to this mode of concerted motion (Van Aalten et al., 1995).

Dynamic cross-correlation map (DCCM) is a correlation map that identifies the correlated (positive or negative) motion between pairs of atoms by examining the magnitude of all the pairwise cross-correlation coefficients (McCammon & Harvey 1988). Herein, we have attempted to present each element of DCCM, in which C*ij* = 1, where the fluctuations of atoms *i* and *j* have the same period and same phase (positively correlated), whereas C*ij* = −1 and C*ij* = 0, respectively, indicate that the fluctuations of *i* and *j* are negatively correlated and not correlated.

#### Free Energy Calculations

One of the endpoint methods that possess an excellent ability to estimate the binding free energy of ligands in the binding site of receptors is the Molecular Mechanics Poisson–Boltzmann Surface Area (MM-PBSA) method (Cheatham et al., 1998; Kuhn & Kollman 2000), which calculates the binding free energy as

$$\Delta {\text{G}}_{\text{binding}}{\;=\text{ G}}_{\text{complex}}\;-\left({\text{G}}_{\text{protein}}{+\text{ G}}_{\text{ligand}}\right)$$ (1)

where the main form of interaction energy can be expressed as

$${\text{G }=\text{ E}}_{\text{MM}}{\;-\text{ TS }+\text{ G}}_{\text{salvation}}$$ (2)

where E_MM_ is the molecular mechanical energy and is defined as

$${\text{E}}_{\text{MM}}{\;=\text{ E}}_{\text{bonded}}{\;+\text{ E}}_{\text{non}-\text{bonded}}{\;=\text{ E}}_{\text{bonded}}\;+\;\left({\text{E}}_{\text{vdw}}{\;+\text{ E}}_{\text{elec}}\right)$$ (3)

where E_bonded_ is a bond angle, is dihedral, and has improper interactions. The non-bonded interactions, i.e., E_non-bonded_ includes both electrostatic (E_elec_) and van der Waals (E_vdw_) interactions, which were modeled using a Coulomb function and a Lennard–Jones potential function, respectively (Cullen et al., 1999). In the MM-PBSA, the energy of transferring the solute from vacuum into the solvent is solvation free energy, which is calculated using an implicit solvent model (Gilson & Honig 1988; Still et al., 1990) as

$${\text{G}}_{\text{salvation}}{\;=\text{ G}}_{\text{polar}}{+\text{ G}}_{\text{non}-\text{polar}}$$ (4)

where G_polar_ and G_non-polar_ are the electrostatic and non-electrostatic contributions to the solvation free energy, respectively.

Furthermore, to obtain the binding free energy of the ligands bound to AChE, g_mmpbsa package (Ma et al., 2002; Kumari et al., 2014) was employed. To estimate the binding energy of each ligand, 150 frames from the last 120-ns MD simulations were extracted to calculate the binding energy and the contribution of each residue in the active sites of AChE.

## Results

### Target Prediction of Huperzine A

**FIGURE 1:**
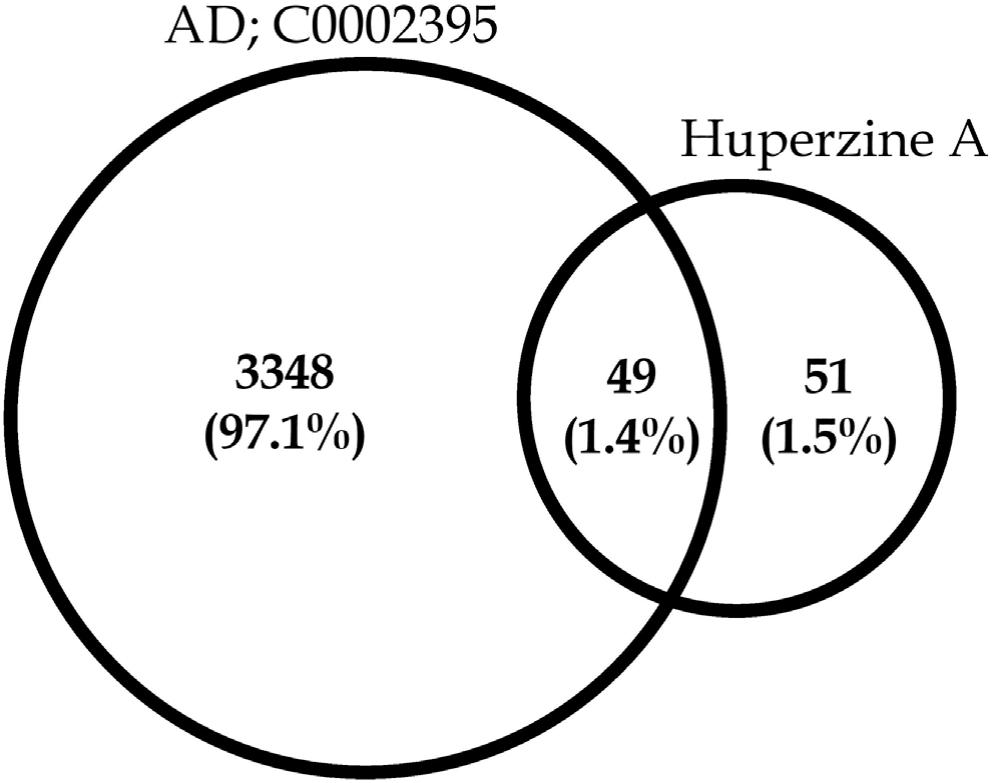
Venn diagram representing huperzine A-regulated proteins count concerning targets related to Alzheimer’s disease (C0002395).

**FIGURE 2:**
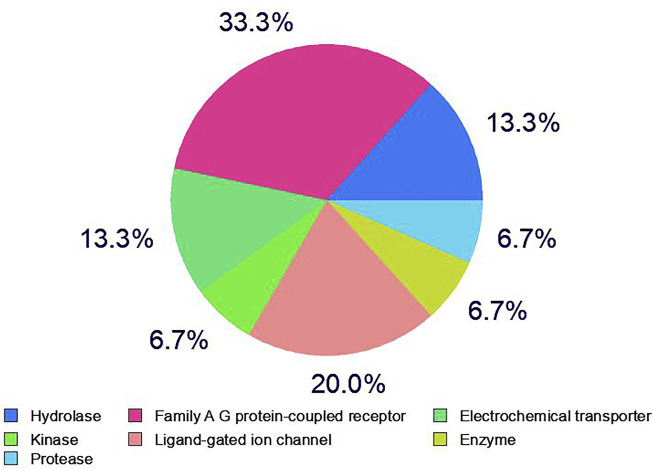
Category of huperzine A-regulated targets in SwissTargetPrediction.

A total of 100 different proteins were predicted as targets of huperzine A, of which 42 were identified to be regulated at a minimum probability of 0.05 in which AChE was considered to be primarily targeted with the probability of 1. Similarly, a total of 49% of the regulated proteins were matched with the reported AD targets (*DisGeNET entry: C0002395*), with a total interaction of 1.4% (Figure 1). Similarly, the huperzine A-regulated targets were under seven different categories, i.e., hydrolase, kinase, protease, family A G protein-coupled receptor, ligand-gated ion channel, electrochemical transporter, and enzyme. Among these, family A G protein-coupled receptors were higher categories, i.e., 33.3% (Figure 2).

### Gene Ontology Analysis

**FIGURE 3:**
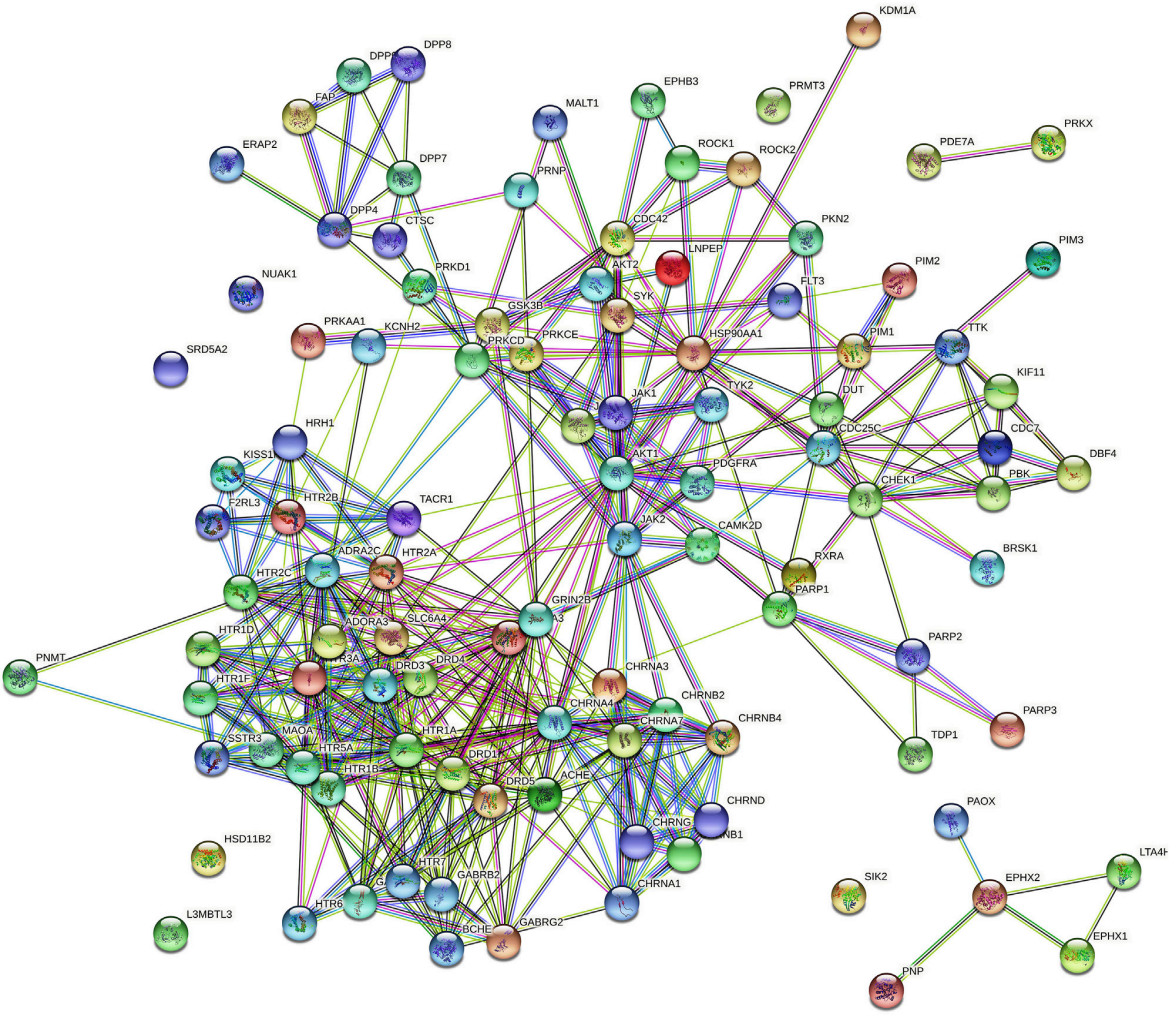
Protein–protein interaction of huperzine A-regulated targets. Node color; 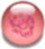
colored nodes: query proteins and first shell of interactors, 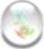
white nodes: second shell of interactors, Node content; 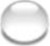
empty nodes: proteins of unknown 3D structure, 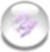
filled nodes: some 3D structure is known or predicted, Known Interactions; 
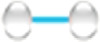
from curated databases, 
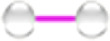
experimentally determined, Predicted Interactions; 
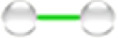
gene neighborhood, 
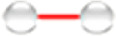
gene fusions, 
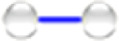
gene co-occurrence and Others; 
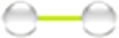
text mining, 
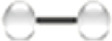
co-expression, 
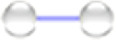
protein homology*.*

A total of 3 Gene Ontology (GO) terms, i.e., cellular components, molecular function, and biological processes, including KEGG pathway(s), were evaluated. The protein–protein interactions of huperzine A-regulated targets are presented in Figure 3, which are based on known interactions (curated databases and experimentally determined), predicted interactions (gene neighborhood, gene fusions, and gene co-occurrence), and miscellaneous (text mining, co-expression, and protein homology).

**FIGURE 4:**
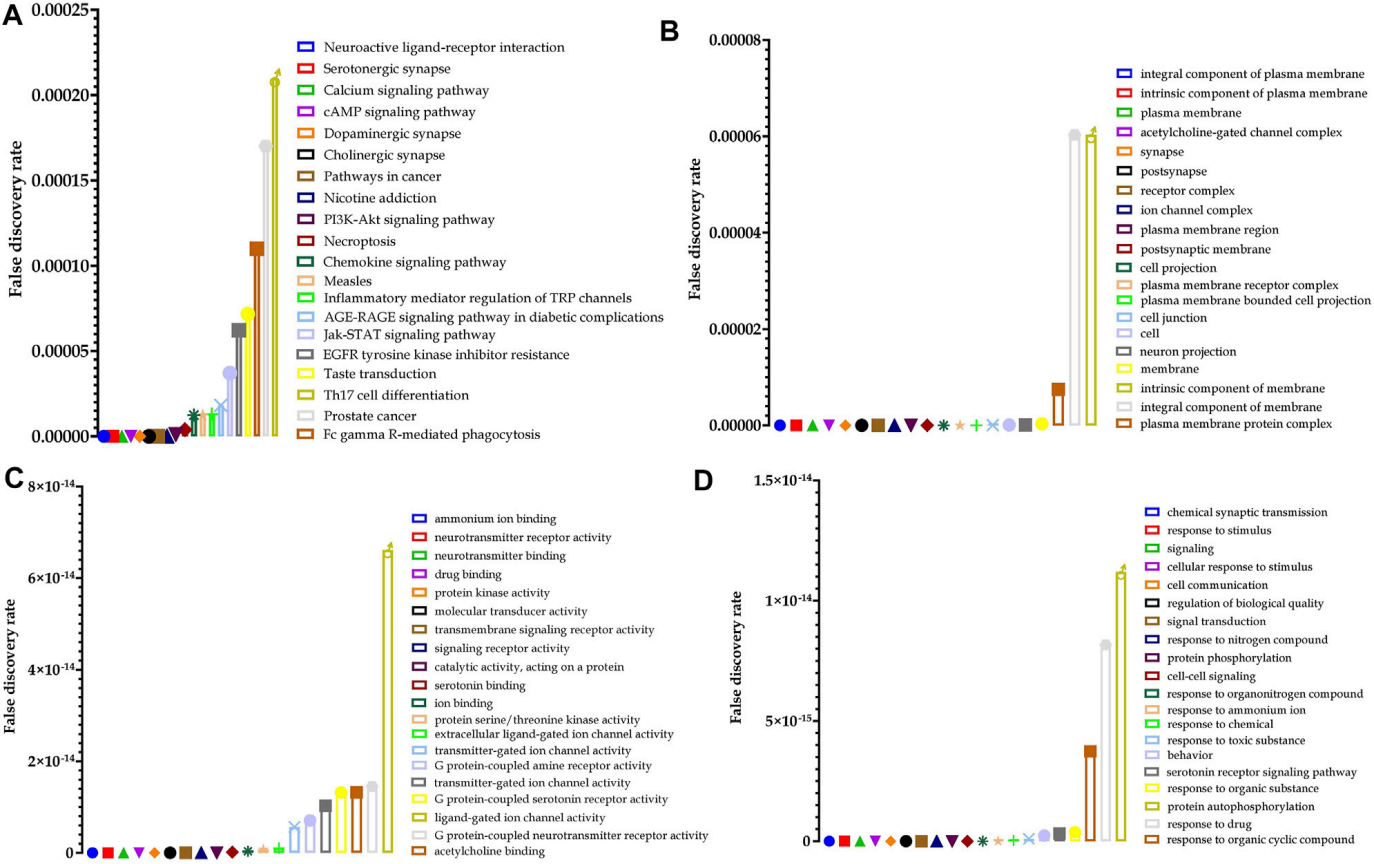
Top 20 huperzine A-regulated **(A)** Kyoto Encyclopedia of Genes and Genomes (KEGG) pathways, **(B)** cellular components, **(C)** molecular function, and **(D)** biological processes along with their false discovery rate.

Furthermore, pathway neuroactive ligand–receptor interaction (*KEGG entry: hsa04080*) was predicted to be primarily modulated *via* regulation of 33 genes (DRD4, KISS1R, F2RL3, CHRND, HTR2B, CHRNA1, CHRNB4, GABRB2, HTR2C, HTR5A, HTR6, TACR1, CHRNB1, DRD5, CHRNA3, HTR1A, HTR1F, HTR7, CHRNB2, HTR1B, CHRNA4, HTR1D, DRD3, CHRNG, DRD1, HRH1, ADRA2C, GABRA1, CHRNA7, GABRG2, HTR2A, GRIN2B, and SSTR3) under 272 background genes, at a false discovery rate of 3.18E−32 among the 101 predicted pathways. Also, a total of 573 genes were modulated in which AKT 1 and 2 appeared in 68 different pathways. The top 20 huperzine A-regulated KEGG pathways are presented in Figure 4.

Similarly, 54 cellular components were predicted to be modulated *via* the regulation of 41 genes (DRD4, LNPEP, KISS1R, FLT3, F2RL3, PDGFRA, CHRND, HTR2B, CHRNA1, SLC6A4, CHRNB4, KCNH2, SLC6A3, GABRB2, HTR2C, HTR5A, HTR6, TACR1, CHRNB1, DRD5, CHRNA3, HTR1A, HTR1F, EPHB3, HTR7, CHRNB2, ADORA3, HTR1B, CHRNA4, HTR1D, DRD3, CHRNG, DRD1, HRH1, ADRA2C, GABRA1, CHRNA7, GABRG2, HTR2A, GRIN2B, and SSTR3) under 1,564 background genes at a false discovery rate of 3.10E−16. Furthermore, 120 molecular functions were identified in which ammonium ion binding was majorly modulated *via* the regulation of 26 (DRD4, CHRND, HTR2B, CHRNA1, SLC6A4, CHRNB4, BCHE, SLC6A3, HTR2C, HTR5A, ACHE, CHRNB1, DRD5, CHRNA3, HTR1A, HTR1F, HTR7, HTR3A, CHRNB2, HTR1B, CHRNA4, HTR1D, DRD3, DRD1, CHRNA7, and HTR2A) genes under 66 background proteins, at a false discovery rate of 4.70E−36. Additionally, a total of 873 biological processes were predicted to be regulated by huperzine A, in which the chemical synaptic transmission was primarily modulated *via* the regulation of 32 genes (DRD4, CHRND, CHRNA1, SLC6A4, CHRNB4, SLC6A3, GABRB2, HTR2C, HTR6, ACHE, TACR1, CHRNB1, DRD5, BRSK1, CHRNA3, HTR1F, GSK3B, HTR7, CHRNB2, HTR1B, CHRNA4, HTR1D, DRD3, CHRNG, DRD1, GABRA1, CHRNA7, GABRG2, HTR2A, AKT1, GRIN2B, and SSTR3) against 402 background genes, at a false discovery rate of 1.67E−24. The 25 hits of GO terms under each category are presented in Figure 4.

### Molecular Docking

**FIGURE 5:**
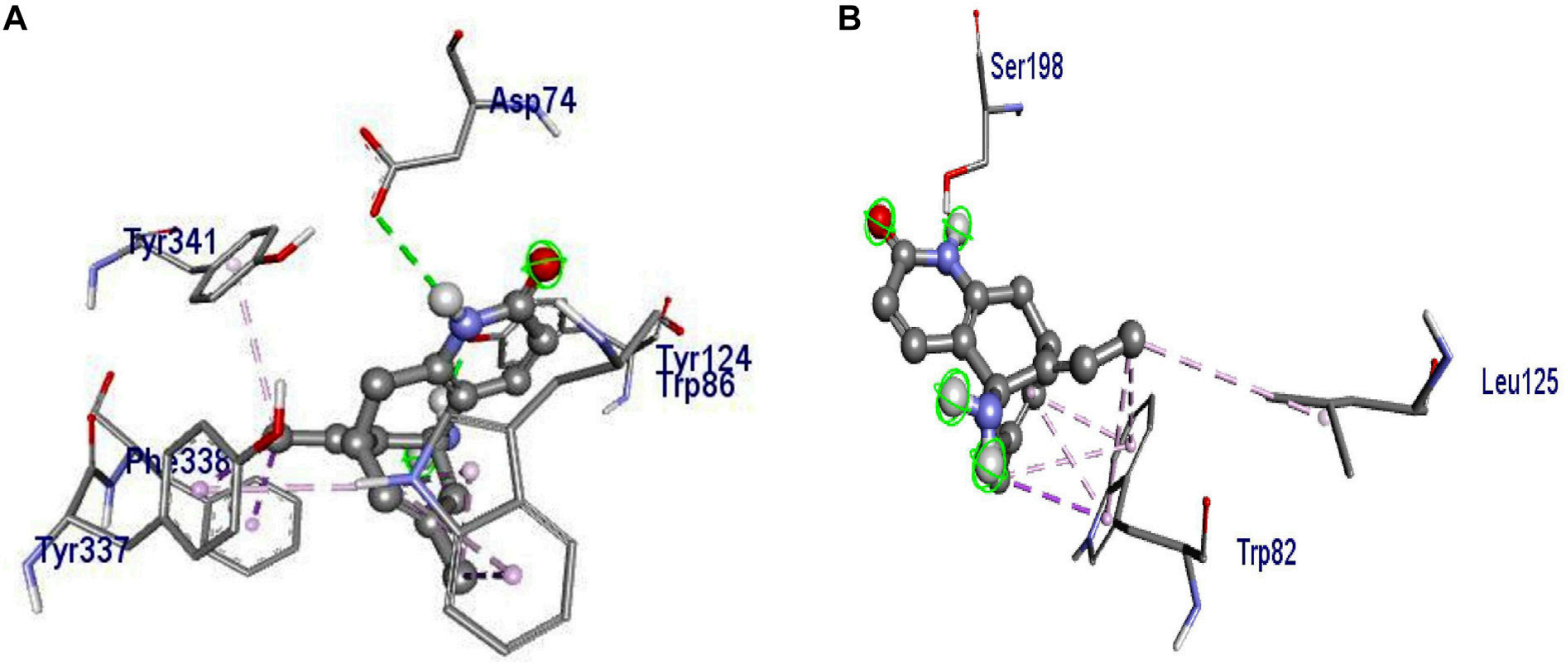
Interaction of **(A)** huperzine A and **(B)** donepezil with acetylcholinesterase (AChE).

Molecular docking revealed that huperzine A possessed binding affinity with AChE (binding energy of −8.7 kcal/mol) *via* 2 H-bond interactions with Tyr382 (22.69) and Ala528 (26.88). Similarly, donepezil showed a binding affinity of −9.4 kcal/mol and had no H-bond interaction but possessed Pi-Pi stacked with Tyr341 (4.35) and Pi-alkyl with Trp286 (5.40) of the benzene and heterocyclic rings, respectively. The interactions of donepezil and huperzine A with AChE are presented in Figure 5.

### Molecular Dynamics Simulations

#### Structural Analysis

**FIGURE 6:**
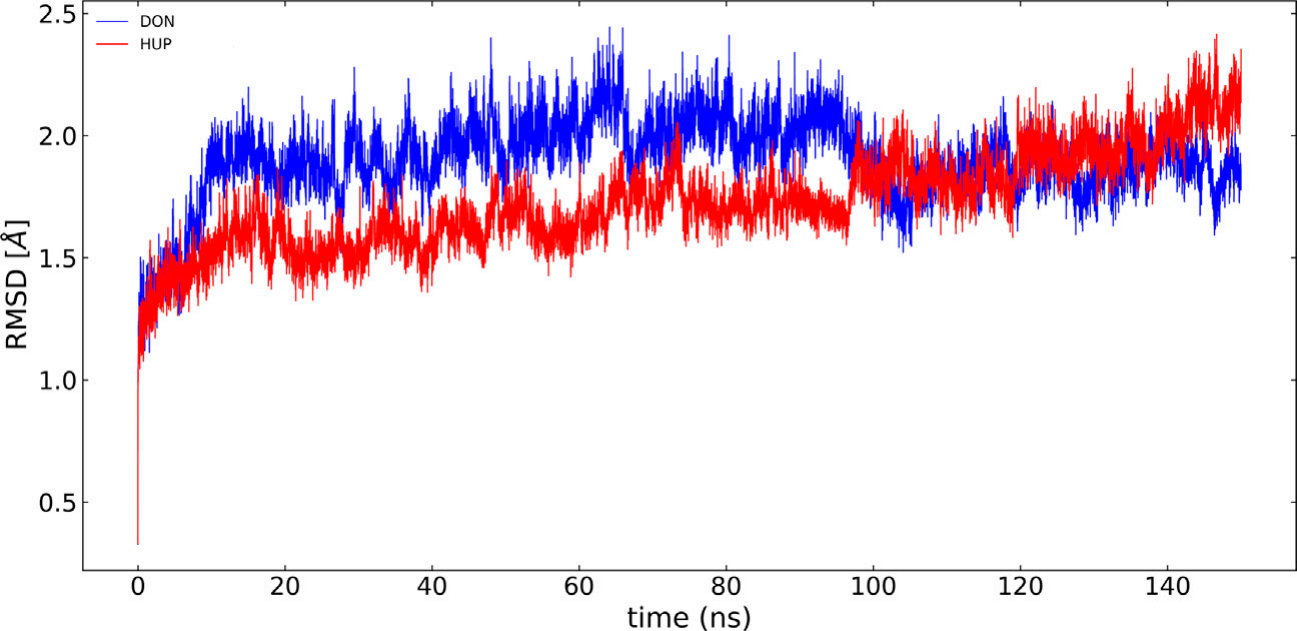
RMSD of acetylcholinesterase (AChE) with donepezil and huperzine A *via* least-square fit to the backbone of starting structure.

**FIGURE 7:**
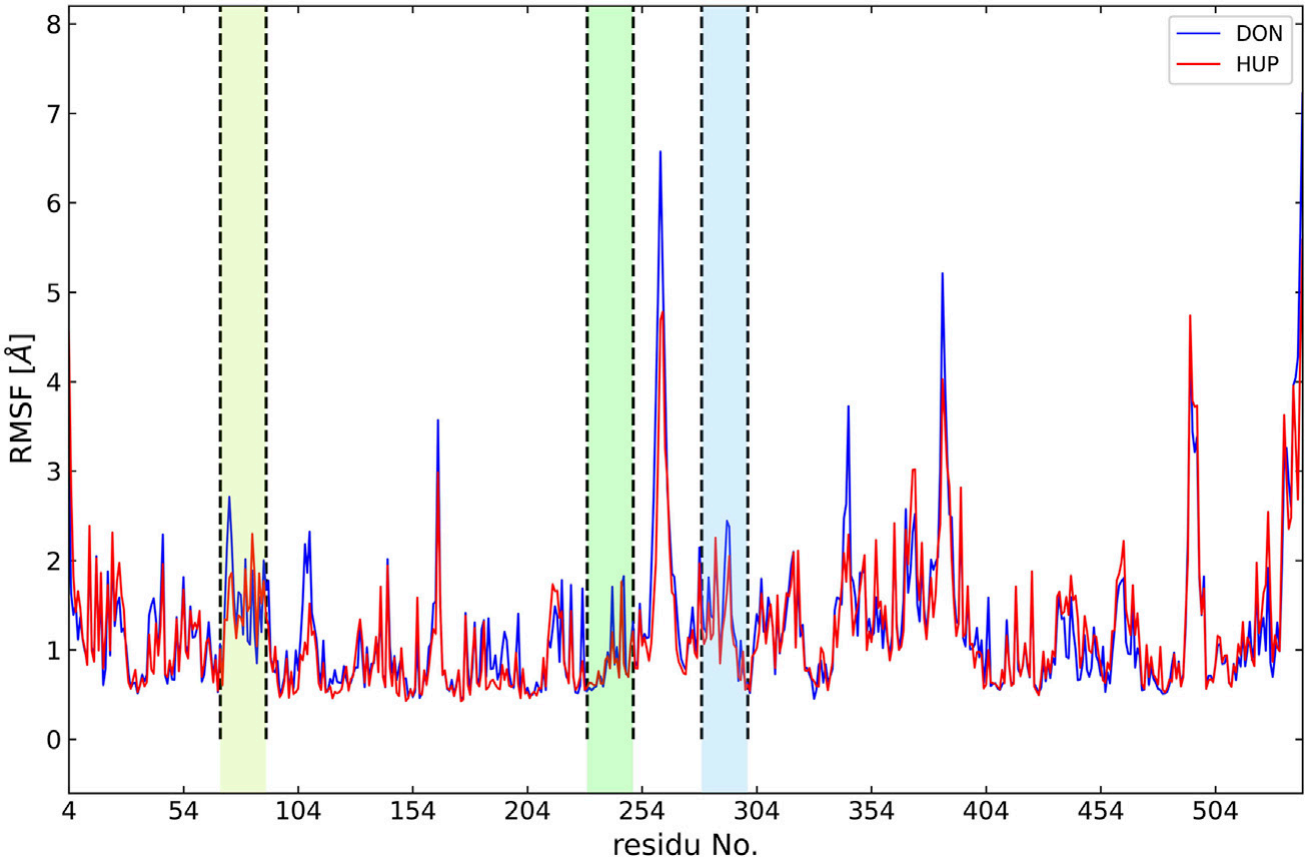
Root-mean-square fluctuation (RMSF) of all molecular dynamics systems throughout 130 ns of simulation as a function of protein residue number. The active site of the protein is depicted as vertical regions.

In the present study, the root-mean-square deviation (RMSD) of Cα atoms of protein backbones was monitored throughout 150-ns MD simulations (Figure 6). The RMSD analysis showed that both ligands, i.e., donepezil and huperzine A, which were bound with AChE, began to relax at ∼20 ns. The RMSD values of AChE-bound ligands fluctuated within 0.5 Å as compared with the docking pose. The root-mean-square fluctuation (RMSF) is defined as the change in the position of every single atom from its average position. The RMSF of MD systems (130-ns simulations) as a function of protein is presented in Figure 7.

**FIGURE 8:**
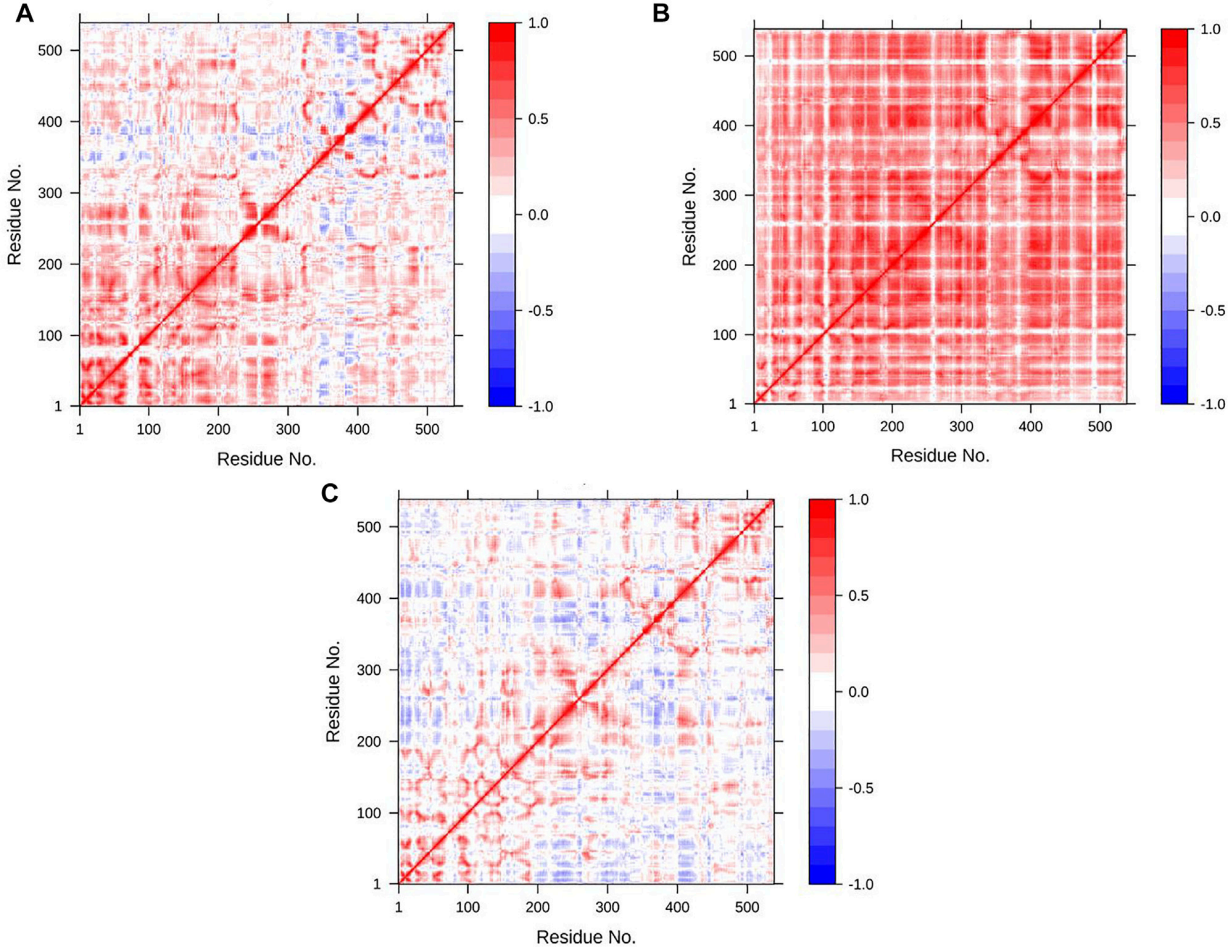
Dynamic cross-correlation map (DCCM) for acetylcholinesterase (AChE) in **(A)** apo state, **(B)** AChE/donepezil, and **(C)** AChE/huperzine A complexes. The positive regions, which are colored in red, are indications of strongly correlated motions of Cα atoms (C*ij* =1), while the negative regions, which are colored blue, are associated with the anticorrelated movements (C*ij* = −1).

One of the descriptive plots obtained from molecular motion is DCCM. This can address the structural motions of an enzyme in its bound and unbound states. However, the PCA is used to construct the correlation matrix for all Cα atoms. It can also be used to assess required eigenvectors to describe the most essential motion of AChE. In our model for DCCM, the first 2 principal components (PCs) were used to generate correlation motion for each structure. Figure 8 illustrates the DCCMs for the unbound state of AChE as well as the bound state with donepezil and huperzine A. In Figure 8, different forms of motions can be observed for the naked state of the enzyme as compared with its bound states. Both donepezil and huperzine A changed the fluctuations of the first 300 residues, including the active site of the enzyme, but donepezil changed all correlated motions into anticorrelated ones when it binds. Huperzine A, on the other hand, changes the anticorrelated motions observed in the apostate into correlated ones, especially in residues 100–300 (Figure 8).

**FIGURE 9:**
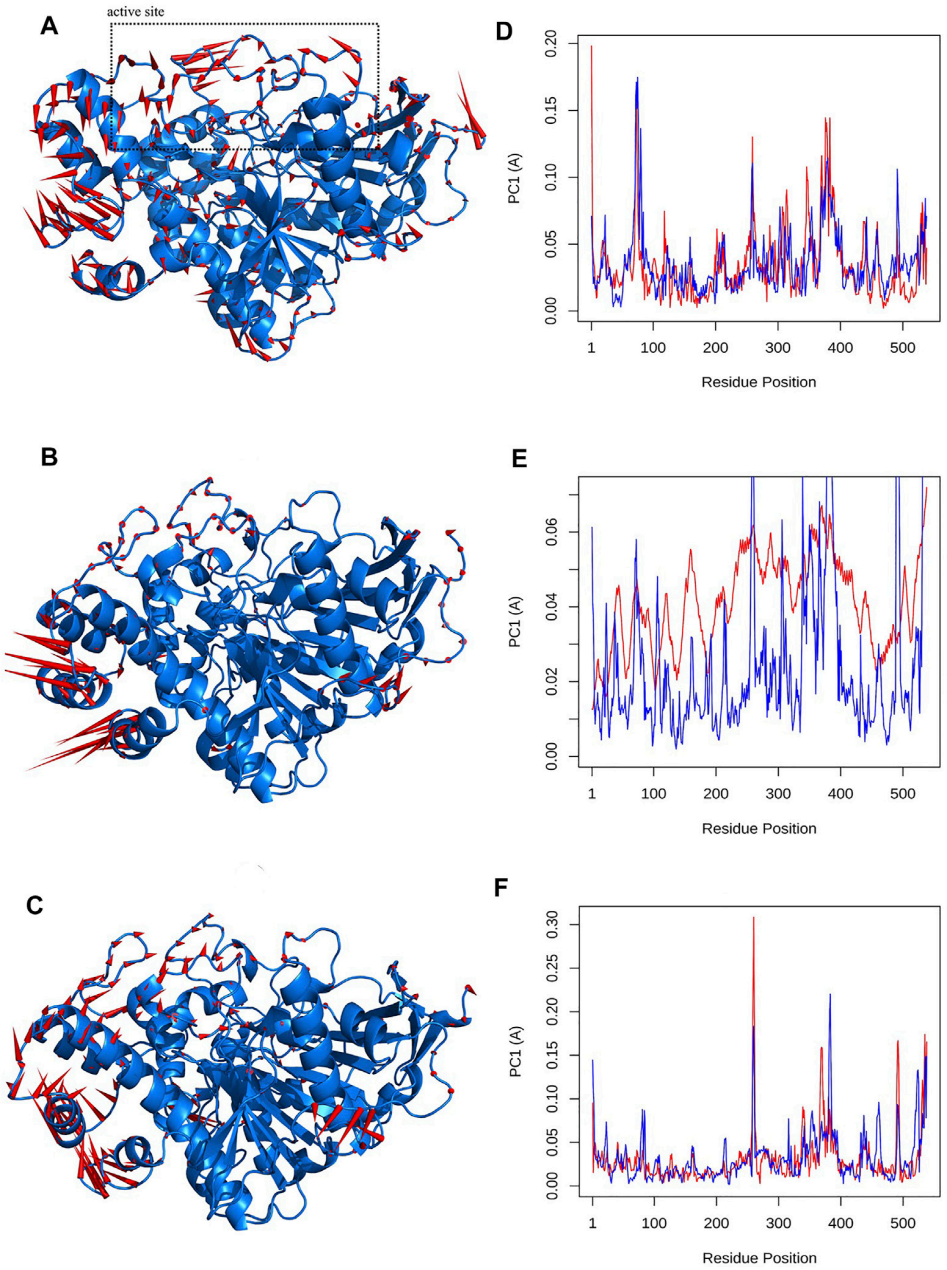
Visualizations of first principal component (PC1) for **(A)** apo state, **(B)** donepezil and, **(C)** huperzine A bound to acetylcholinesterase (AChE). The Q20 residue-wise plots for PC1 (red) and PC2 (blue) for **(D)** AChE in Q20 apo state. **(E)** AChE–donepezil and **(F)** AChE–huperzine A are also represented.

**FIGURE 10:**
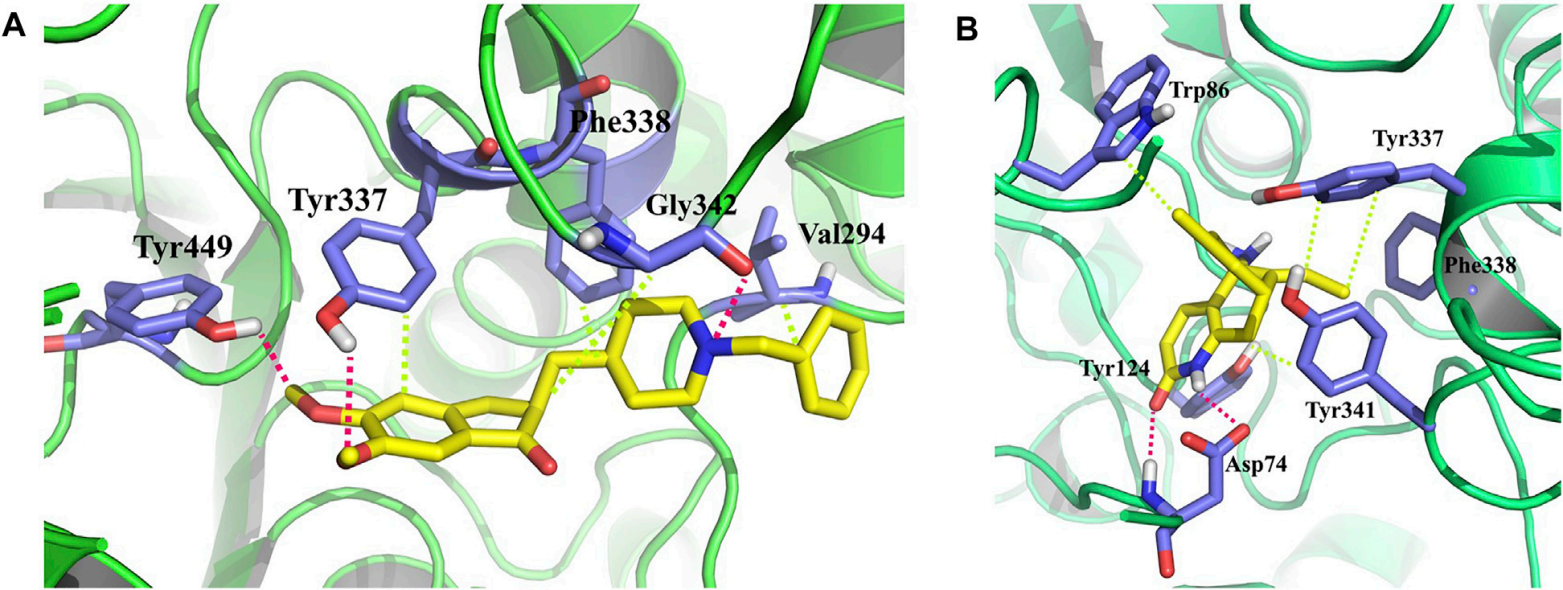
3D representation of representative structures of **(A)** donepezil and **(B)** huperzine A in the active site of acetylcholinesterase (AChE). Hydrophobic interaction is depicted as lime dash lines, whereas the H-bond is illustrated as a red dash line.

Besides the correlation map analysis, which depicts the movements of every atom of the protein, one needs to inspect the overall collective motions that are responsible for the dominant protein’s conformational changes. This can be achieved *via* the projection of conformations onto the orthogonal collective motions (eigenvectors) output of PCs. In Figure 9, the first main PC (PC1) for protein bound to the inhibitors and its apo form is presented. The vector field representation in PyMOL was used to present the direction and magnitude of each Cα atom in PC1. In the enzyme apo form, the large-magnitude motions were observed within the amino acid residues of the active site of the protein (depicted as rectangles in Figure 9A), primarily belonging to the loops. However, these collective motions vanished in the presence of donepezil and huperzine A, indicating the role of loops in the overall movement of the protein and its function. In Figures 9 (D–F), the contribution of each residue to the PC1 (red) and PC2 (blue) for protein in bound and unbound states is depicted. As depicted in Figure 9D, protein adopts a large magnitude of motion within the active site to the protein wherein donepezil and huperzine A are predicted to bind (residues 70–90 and 360–400). However, these movements were drastically diminished when donepezil (Figure 9E) and huperzine A (Figure 9F) bound to the enzyme. In addition, dominant interactions to stabilize donepezil were within the active site of AChE H-bonds, through the phenolic and piperidine moieties with Tyr449, Tyr337, and Gly342 (Figure 10A). Also, huperzine A seemed to act within the active site of AChE to have an H-bond with Asp74 and 2 hydrophobic interactions with Thr337 and Tyr124 (Figure 10B).

#### H-Bond Analysis

To evaluate quantitatively the firmness of H-bonds between donepezil and huperzine A with AChE, MD analysis of ligand–AChE stability was monitored during the 150 ns of the trajectory period. The threshold for H-bond was 3.5 Å with 30 ∠ (different from the PLIP server mentioned above), as summarized in Table 1. Furthermore, huperzine A possessed strong H-bonds with the amino acid residues within the active site of AChE. The higher occupancies regarding O1 and N2 atoms in huperzine A involved H-bonds with Asp74 and Ser125. The percent of occupancy showed the number of frames in which the particular H-bond had been monitored, out of all the processed frames. Also, donepezil interacted with residues in the active site of AChE occupying Tyr124, Tyr337, and Ser198.

**TABLE 1:** H-bond analysis of donepezil and huperzine A with AChE.

Compounds	H-Bonds		
	Acceptor atom	Donor atom	% Occupancy
Donepezil	Donepezil @ O1	Tyr124 @ OH	6.42
	Donepezil @ O27	Tyr337 @ OH	1.41
	Donepezil @ O1	Ser198 @ OG	0.57
Huperzine A	Huperzine A @ O1	Asp74 @ N	33.11
	Asp74 @ OD1	Huperzine A @ N2	23.49
	Asp74 @ OD2	Huperzine A @ N2	18.19
	Huperzine A @ O1	Ser125 @ OG	9.33

Note. AChE, acetylcholinesterase.

#### Binding Free Energy and Energy Decomposition Analysis

The binding affinity of both the ligands was evaluated based on the binding free energy using the MM-PBSA method. Table 2 summarizes the details of the contribution in terms of the binding free energies for the inhibitors as defined by Eqs 1–4. The binding affinity of donepezil with AChE was observed to be higher as compared with huperzine A.

**TABLE 2:** Free energy of donepezil and huperzine A with AChE.

	AChE	
	Donepezil	Huperzine A
Van der Waals	−42.06 ± 7.06	−26.47 ± 1.97
Electrostatic	−2.10 ± 1.48	−7.89 ± 3.53
Polar salvation	21.92 ± 5.76	18.73 ± 4.92
SASA energy	−4.85 ± 0.81	−3.21 ± 0.17
Binding energy	−27.13 ± 4.52	−18.85 ± 3.76

Note. All energies in the table are in kcal/mol.

AChE, acetylcholinesterase.

Furthermore, it was observed that Van der Waals’s energy strongly favored the stability of donepezil within the active site of AChE. The electrostatic energy favored just the huperzine A ligand in AChE protein, while the SASA energy asserted almost the same effect on both ligands.

**FIGURE 11:**
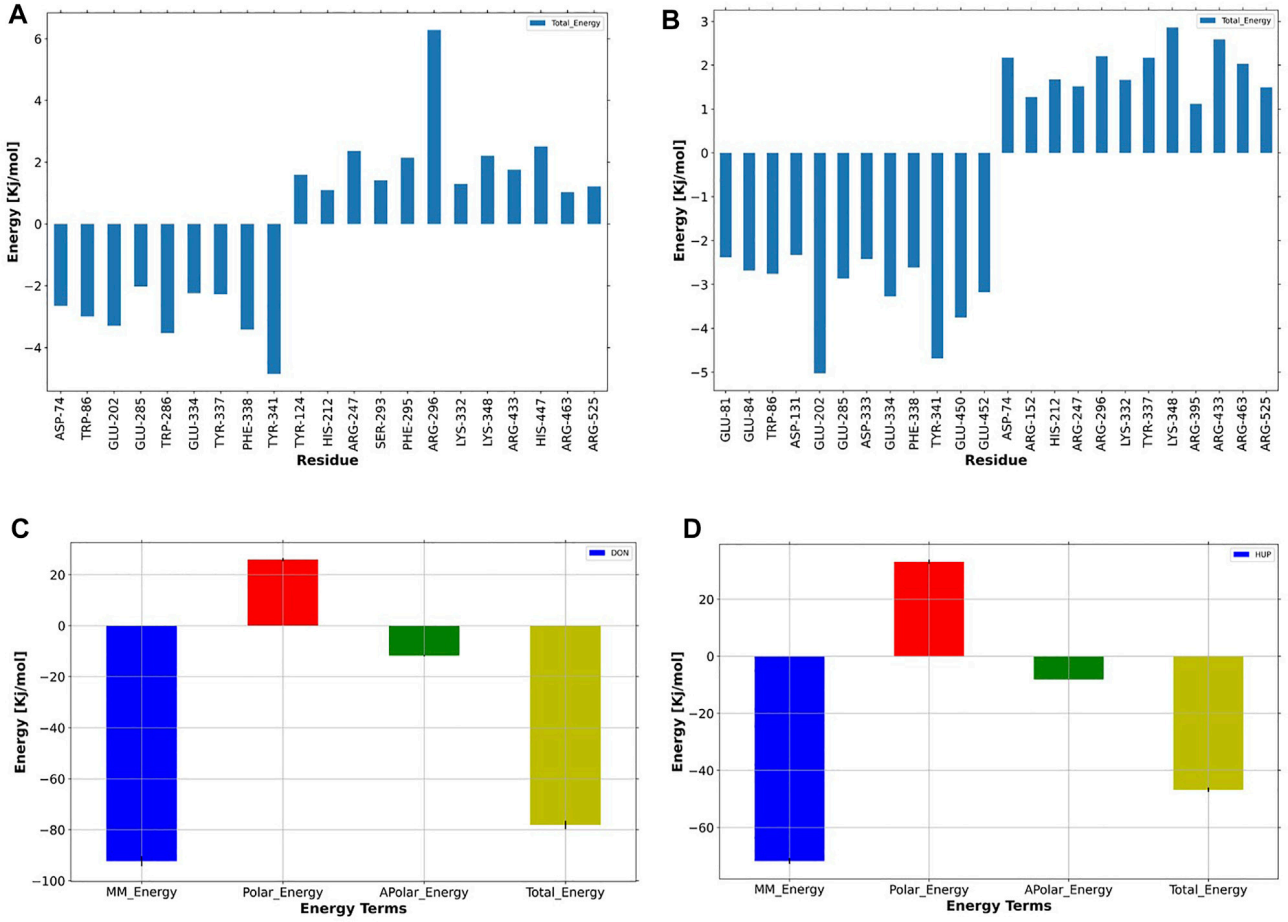
Per-residue binding energy decomposition of predicted **(A)** donepezil/acetylcholinesterase (AChE) and **(B)** huperzine A/AChE. The energy contribution larger than ±1 kcal/mol is displayed. The blue bars show the residues with an absolute binding free energy. Energy decomposition of each ligand versus energy terms depicted for **(C)** donepezil and **(D)** huperzine A, where MM, polar, nonpolar, and total energies of ligand contribution to the binding free energies are colored as dark-blue, red, green, and yellow, respectively.

Another practical statistic is that the g_mmpbsa tool helps to discover the reciprocal contribution of each residue to the binding free energy of AChE–ligand complexes, based on the dynamic of systems, as depicted in Figure 11. In the case of donepezil, decomposition analysis revealed that Tyr341, Phe338, Trp286, Glu202, and Asp74 had a considerable contribution to the binding energy of this ligand within the active site of AChE. Also, Glu202, Tyr341, Glu450, Glu452, Glu334, and Phe338 favored the binding energy of huperzine A.

## Discussion

The present study utilized a series of system biology tools to evaluate the efficacy of huperzine A against AD. Initially, huperzine A-modulated targets were identified, which were later enriched in STRING. Furthermore, huperzine A was also docked against AChE, as it was predicted to be majorly modulated with the highest probability, followed by MD simulations.

AChE has been identified as the major target against AD, on which multiple selective or non-selective AChE inhibitors have been established (Russ and Morling, 2012), which check the hydrolysis of acetylcholine. Also, AChE deregulation has been demonstrated in multiple neurodegenerative pathogeneses (Mushtaq et al., 2014). Thus, AChE inhibition may help in managing the pathogenesis of AD. Therefore, the present study investigated the probability and potential of huperzine A to act over AChE.

Though the concept of “lock and key” is an established approach to develop new therapeutic agents against various diseases, it is to be understood that a “single compound may regulate multiple proteins” with the concept of “master key can unlock multiple locks” (Patwardhan 2014; Duyu et al., 2021; Khanal & Patil 2021), in which the master key can be conceptualized for its broad-spectrum pharmacological activity. Based on this theory, huperzine A-regulated targets were enriched, which identified a total of 101 different pathways. Among them, neuroactive ligand–receptor interaction was predicted to be majorly modulated, with the least false discovery rate. Overall, two main proteins, i.e., Akt 1 and 2, were identified to be common in a total of 68 multiple pathways.

KEGG pathway classifies neuroactive ligand–receptor interaction (KEGG entry: hsa04080; https://www.genome.jp/dbget-bin/www_bget?hsa04080) as an environmental information processor and also contributes to the interaction of signaling molecules, based on which multiple centrally acting drugs over serotonin, dopamine, adrenergic, and cell surface glycoproteins are established. Furthermore, its ortholog presents the association of this pathway (KEGG entry: ko04080; https://www.genome.jp/dbget-bin/www_bget?ko04080) with muscarinic, adrenergic, dopamine, histamine, serotonin, gamma-aminobutyric acid (GABA), nicotinic, and glutamine receptors, which have a direct or indirect contribution in various neurodegenerative pathogeneses. Thus, it may be assumed that the efficacy of huperzine A to modulate these surface receptors can be utilized against AD, as the approach has been approved to target the above-mentioned proteins in dealing AD. Additionally, serotonergic synapse (KEGG entry: hsa04726; https://www.genome.jp/dbget-bin/www_bget?pathway+hsa04726), cholinergic synapse (KEGG entry: hsa04725; https://www.genome.jp/entry/pathway+hsa04725), and nicotine addiction (KEGG entry: hsa05033; https://www.genome.jp/dbget-bin/www_bget?hsa05033) are also identified, which are directly involved in the progression of AD pathogenesis.

Furthermore, GO analysis outlined the multiple terms concerning the various neurodegenerative diseases, including AD. In the cellular components, it was identified that the acetylcholine-gated channel complex (*GO:0005892*) and the GABA-A receptor complex (*GO:1902711*) may get regulated *via* huperzine A-regulated protein–protein interaction. In AD, there is a malfunction of the surface receptor(s) and an increase in β-amyloid deposition (Murphy and LeVine, 2010). In the present study, huperzine A-regulated proteins showed efficacy to trigger the neurotransmitter receptor activity (*GO:0030594*), neurotransmitter binding (*GO:0042165*), G protein-coupled serotonin receptor activity (*GO:0004993*), acetylcholine binding (*GO:0042166*), G protein-coupled neurotransmitter receptor activity (*GO:0099528*), and β-amyloid binding (*GO:0001540*) and could thus ameliorate AD.

Owing to the complex pathogenesis of AD, attributed to the deregulation of multiple signaling pathways (Calabrò et al., 2020), it has been categorized as a polygenic pathogenesis (Bird 2008). Previously, decreased memory (Jahn 2013), altered β-amyloid response (Rauch et al., 2011), and deregulated serotonin (Ouchi et al., 2009), dopamine (Pan et al., 2019), and choline pathways (Whiley et al., 2014) had been linked with AD. In the present study, we observed the regulation of serotonin receptor signaling pathway (*GO:0007210*), cholinergic synaptic transmission (*GO:0007271*), memory (*GO:0007613*), dopamine metabolic process (*GO:0042417*), acetylcholine receptor signaling pathway (GO:0095500), and response to β-amyloid (*GO:1904645*) as the multiple biological spectra against AD.

AChE has been reported to be the major target to manage AD, based on which multiple cholinesterase inhibitors, including donepezil, were established (Mushtaq et al., 2014). Inhibitors of these enzymes monitor the AChE function (Nordberg et al., 2013; Mushtaq et al., 2014) and regulate the hydrolysis of acetylcholine. In target prediction, huperzine A was identified to act primarily over AChE. Hence, the independent action of huperzine A over AChE was evaluated *via* molecular docking, which showed that the binding affinity of huperzine A was almost nearer to a clinically practiced gold standard cholinesterase inhibitor (donepezil). Thus, it was further corroborated with MD simulation evaluation.

Structural analyses, such as RMSD and RMSF, are efficient tools to examine the molecular interaction effectiveness of molecules (Khanal et al., 2021). In the MD simulations, the dynamics of AChE in apo forms were altered, as compared with the bound states, due to the binding site occupation by the ligands whose illustrations reflected the swinging of the docking pose within 0.5–0.7 Å for the complex indicating the docking poses to be reliable. Furthermore, clustering analysis is chiefly used to discover protein conformations by diminishing the size of the problem of conformational analysis by dividing the conformations gathered into separate groups (Knaggs et al., 2007). The mid-point structure is commonly used to establish the conformations, within a specific cluster to secure the cluster representative, which is a physically sensible structure (Knaggs et al., 2007). Conformational clustering was performed on the trajectories obtained from the MD simulations of the ligand–AChE complex to select the representative conformations for further experimentation. Clustering was performed using Cα backbone atoms, least-squares alignment, and the Gromos algorithm (Daura et al., 1999), at a cutoff of 0.15 nm, by the g_cluster module as implemented in Gromacs 2020.2. However, the center structures were the most populated. Hence, the more stable clusters of each complex were considered to be representatives of the binding mode conformations.

It was observed that two factors, i.e., H-bond and hydrophobic forces, were important for the stabilization of ligands within the active sites of AChE, as showed by the donepezil–AChE stabilization *via* the hydrogen and hydrophobic interactions. Similarly, the huperzine A–AChE complex was also observed within the same active site, stabilized *via* hydrogen and hydrophobic interactions.

Further, it was observed that the binding energy in molecular docking and MD simulations of both the ligands showed that donepezil had a higher binding affinity as compared with huperzine A. However, not limiting to AChE inhibition, huperzine A was predicted to be involved with regulating multiple pathways, and biological processes and functions that are related to AD.

## Conclusion

The study revealed that huperzine A regulated the multiple proteins and pathways concerning AD. Also, it revealed the probability to act over various neurotransmitter synapse and metabolic pathways, other than the direct action over the AChE. Since the present outcomes are derived from multiple computational approaches, the findings need to be validated *via* the *in vitro* and *in vivo* experimental approaches. Thus, the study not only establishes the potential of huperzine A in AD through *in silico* studies but also provides a lead to the modification of the structure of huperzine A to enhance its binding affinity with AChE.

## Data Availability

The raw data supporting the conclusions of this article will be made available by the authors, without undue reservation.
